# A Chaperone-Like Role for EBI3 in Collaboration With Calnexin Under Inflammatory Conditions

**DOI:** 10.3389/fimmu.2021.757669

**Published:** 2021-09-17

**Authors:** Aruma Watanabe, Izuru Mizoguchi, Hideaki Hasegawa, Yasuhiro Katahira, Shinya Inoue, Eri Sakamoto, Yuma Furusaka, Ami Sekine, Satomi Miyakawa, Fumihiro Murakami, Mingli Xu, Toshihiko Yoneto, Takayuki Yoshimoto

**Affiliations:** Department of Immunoregulation, Institute of Medical Science, Tokyo Medical University, Tokyo, Japan

**Keywords:** EBI3, calnexin, chaperone, heterodimer, tumor growth

## Abstract

The interleukin-6 (IL-6)/IL-12 family of cytokines plays critical roles in the induction and regulation of innate and adaptive immune responses. Among the various cytokines, only this family has the unique characteristic of being composed of two distinct subunits, α- and β-subunits, which form a heterodimer with subunits that occur in other cytokines as well. Recently, we found a novel intracellular role for one of the α-subunits, Epstein-Barr virus-induced gene 3 (EBI3), in promoting the proper folding of target proteins and augmenting its expression at the protein level by binding to its target protein and a well-characterized lectin chaperone, calnexin, presumably through enhancing chaperone activity. Because calnexin is ubiquitously and constitutively expressed but EBI3 expression is inducible, these results could open an avenue to establish a new paradigm in which EBI3 plays an important role in further increasing the expression of target molecules at the protein level in collaboration with calnexin under inflammatory conditions. This theory well accounts for the heterodimer formation of EBI3 with p28, and probably with p35 and p19 to produce IL-27, IL-35, and IL-39, respectively. In line with this concept, another β-subunit, p40, plays a critical role in the assembly-induced proper folding of p35 and p19 to produce IL-12 and IL-23, respectively. Thus, chaperone-like activities in proper folding and maturation, which allow the secretion of biologically active heterodimeric cytokines, have recently been highlighted. This review summarizes the current understanding of chaperone-like activities of EBI3 to form heterodimers and other associations together with their possible biological implications.

## Introduction

**Figure 1:**
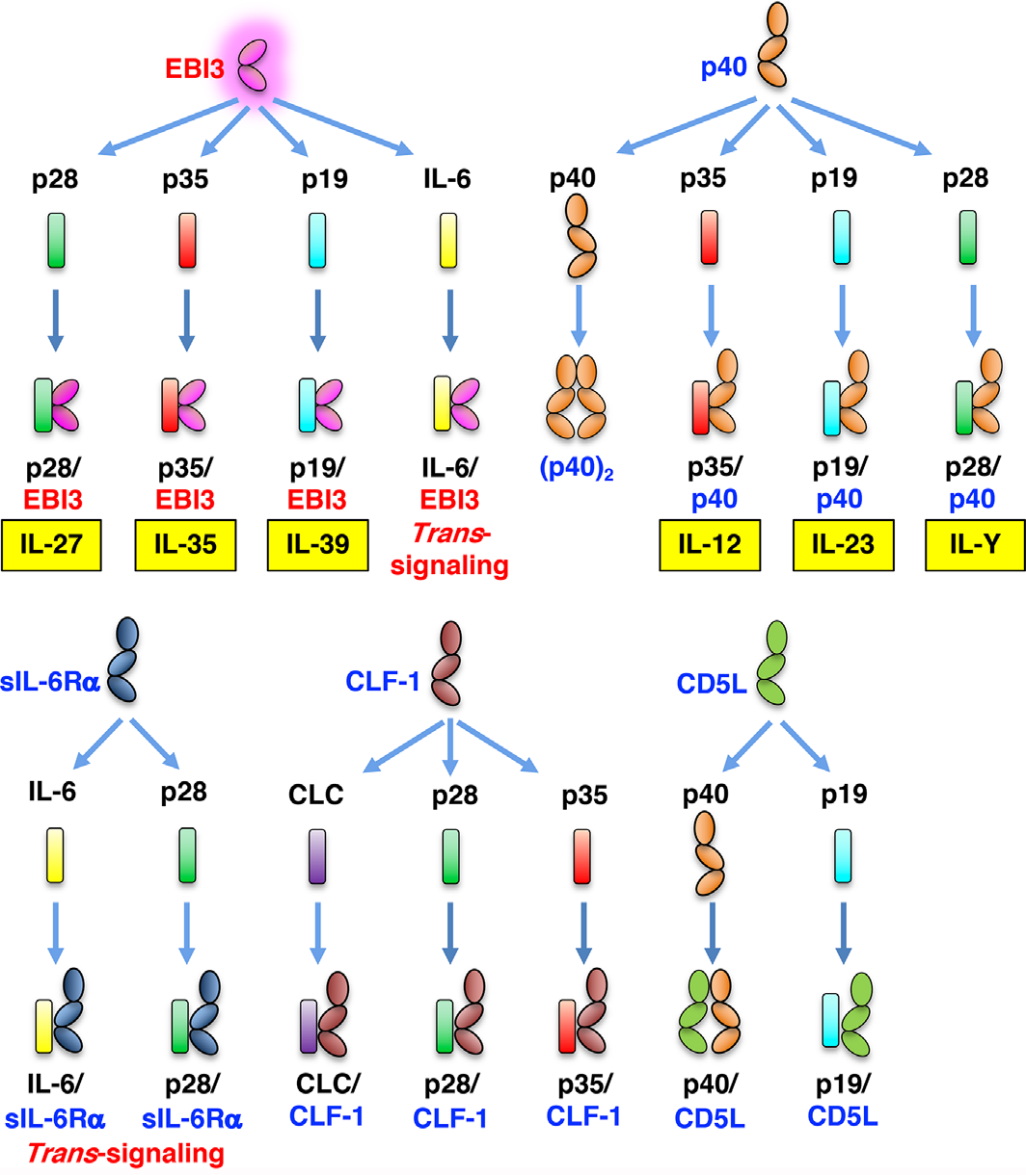
Expanding diversity of the IL-6/IL-12 heterodimeric cytokines and their related heterodimeric molecules. Analogous to the complex of IL-6 and soluble IL-6Rα (IL-6/sIL-6Rα), which induces IL-6 trans-signaling in gp130-expressing cells, various heterodimeric cytokines have been reported and their number is still increasing. CLC, cardiotrophin-like cytokine.

The interleukin-6 (IL-6) family of cytokines was originally defined as cytokines that utilize the common signaling receptor subunit β-receptor glycoprotein 130 (gp130) and comprise IL-6, IL-11, oncostatin M, leukemia inhibitory factor, ciliary neurotrophic factors, cardiotrophin 1, and cardiotrophin-like cytokine factor 1 (1). IL-6 has two very unique signaling pathways: IL-6 classic signaling and IL-6 trans-signaling. IL-6 classic signaling is generally through the receptor complex of membrane-bound IL-6Rα and gp130. By contrast, IL-6 trans-signaling occurs under pathological conditions, in which the soluble form of IL-6Rα (sIL-6Rα) is generated and IL-6 bound to it (IL-6/sIL-6Rα) signals through gp130. The IL-6/sIL-6Rα complex is similar to the IL-12 family of cytokines in molecular structure in that it is a heterodimeric glycoprotein consisting of two distinct subunits, the α- and β-subunit; the α-subunit includes IL-12p35, IL-23p19, and IL-27p28, and the β-subunit includes IL-12p40 and Epstein-Barr virus (EBV)-induced gene 3 (EBI3) (2). Therefore, the IL-6 and IL-12 families of cytokines together are called the IL-6/IL-12 family. This suggests that six potential heterodimers can be formed: IL-12 (p35/p40) (3, 4), IL-23 (p19/p40) (5), IL-27 (p28/EBI3) (6), IL-35 (p35/EBI3) (7), and IL-39 (p19/EBI3) (8–10) are bioactive, and p28/p40, which is putatively called IL-Y (**Figure 1**) (11). The subunits of IL-12, IL-23, and IL-Y are connected by a disulfide bridge with each subunit, whereas the subunits of IL-27 and IL-35 are not. As receptor subunits, IL-12 and IL-23 utilize the receptor complex between IL-12 receptor β1 (IL-12Rβ1) (12) and IL-12Rβ2 (13), and IL-12Rβ1 (5) and IL-23Rα (14), respectively. IL-27 and IL-Y signal *via* the receptor complexes between gp130 and the IL-27 receptor (WSX-1) (15) and possibly between IL-12Rβ1 and WSX-1, respectively, although the latter complex has not yet been proven (11). For IL-35, four receptor complexes have been described: IL-12Rβ2/gp130, IL-12Rβ1/WSX-1, IL-12Rβ2/IL-12Rβ2, and gp130/gp130 (16). For IL-39, the receptor complex between IL-23Rα and gp130 has been reported (10). Of note, by shuffling the extracellular and intracellular domains of the IL-6/IL-12 cytokine receptors, synthetic IL-35 and IL-39 signaling was demonstrated to be biologically active (17). Interestingly, these analyses further identified additional biologically active synthetic receptor combinations, IL-12Rβ1/gp130 and IL-23Rα/IL-12Rβ2, whose ligands have not yet been identified (17). As downstream signaling molecules, Janus kinase (JAK)/signal transducer and activator of transcription (STAT) play critical roles in the induction of respective cellular responses. IL-12 and IL-23 mainly activate STAT4 (18) and STAT3 (14), respectively, whereas IL-27 and IL-39 mainly activate STAT1 and STAT3, respectively (9, 19, 20). By contrast, IL-35 mainly activates both STAT1 and STAT4 (16).

Of note, the diversity in molecular structures and functions of the IL-6/IL-12 family of cytokines and their related heterodimeric molecules [e.g., p28/sIL-6Rα (21), p28/cytokine-like factor-1 (CLF-1) (22), p35/CLF-1 (23), IL-6/EBI3 (24), p40/CD5L (25), p19/CD5L (26); **Figure 1**] are still growing (2). For proper folding and maturation to form such bioactive and secreted heterodimeric cytokines, their chaperone-like activities have been recently highlighted (27–31). In this review, we summarize and discuss recent advances on the possible molecular mechanisms underlying the generation of such diversity *via* the chaperone-like functions of EBI3 and possibly p40.

## Ability of EBI3 to Associate With Other Proteins

EBI3 was originally identified as an inducible cytokine receptor-like molecule homologous to p40 in B lymphocytes after EBV infection (32). EBI3 is a 34 kDa glycoprotein that contains a signal peptide and two fibronectin type III domains but lacks a membrane-anchoring motif; it is secreted and is also present on the plasma membrane and endoplasmic reticulum (ER) (32). Although the original cloning paper (32) demonstrated that EBI3 associates with calnexin (33, 34), p62 (sequestone-1 or SQSTM1) (35, 36), and an uncharacterized protein (called p78), the physiological significance of these interactions remains unknown, with the exception of calnexin as mentioned below (27). Calnexin plays a critical role in assisting a quality control ensuring proper folding of proteins destined for the plasma membrane or secretion through binding to partially folded or misfolded proteins (34). Calnexin contains a lectin site that recognizes substrate proteins and by this interaction calnexin binds to and participates in the folding of most if not all glycoproteins including MHC class I molecules (33). Calnexin recruits enzymes that catalyze disulfide bond formation and isomeriztion to aid in folding. Calnexin binding retains substrate proteins in the ER until they are fully matured or, for terminally misfolded proteins, until they are targeted for degradation. p62 is a multi-domain and multi-functional adaptor protein, representing not only a selective cargo receptor for autophagy thus inhibiting inflammasome activation and inflammation, but also a central signaling hub, linking several important pro- and anti-inflammatory pathways (35, 36). By binding to the Nrf2 inhibitor Keap1 and resultant autophagosomal degradation, p62 activates Nrf2 and attenuates inflammation. Moreover, p62 activates the central kinase mTOR of the mTORC1 sensor complex that controls cell proliferation and differentiation, and also activates NF-κB, an important regulation of inflammation and cancer development. Subsequently, EBI3 was shown to associate with p35 to form a novel heterodimer p35/EBI3 (37), but the functions of this complex remained unknown until 2017, when their immunosuppression functions were clarified and the composite molecule was named IL-35 (7).

## EBI3 Is Necessary for the Proper Folding of IL-23Rα *via* the Chaperone Calnexin

**Figure 2:**
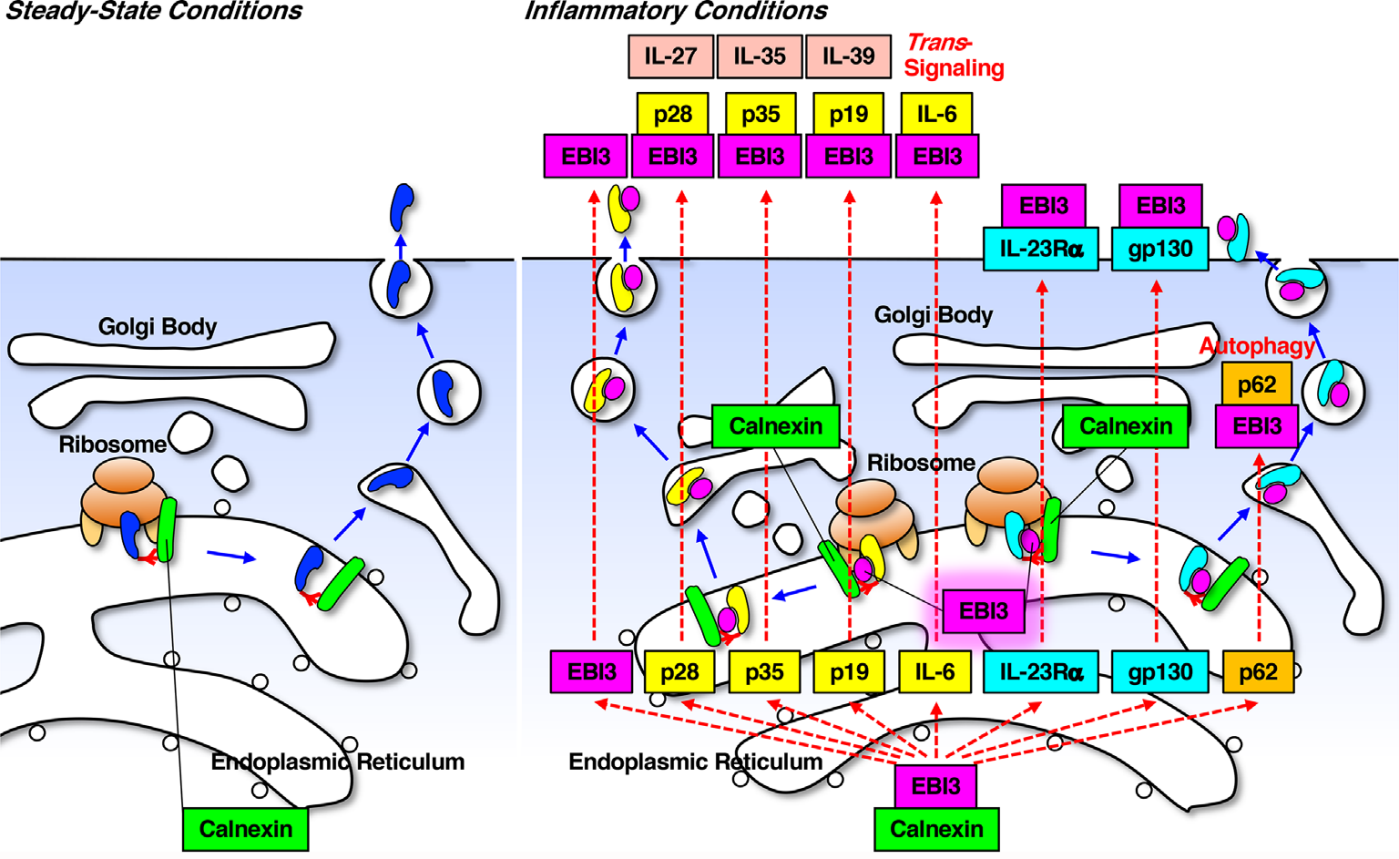
EBI3 plays an important role in promoting the formation of proper protein folding in collaboration with calnexin under inflammatory conditions. Calnexin plays a critical role in assisting a quality control ensuring proper folding of most if not all glycoproteins including MHC class I molecules destined for the plasma membrane or secretion by binding to partially folded or misfolded proteins. p62 is a multi-domain and multi-functional adaptor protein, representing not only a selective cargo receptor for autophagy thus inhibiting inflammasome activation and inflammation, but also a central signaling hub, linking several important pro- and anti-inflammatory pathways including Nrf2, mTOR, NF-κB, and so on. Because calnexin expression is constitutive but EBI3 expression is inducible through activation of TLRs and T-cell receptors, calnexin plays a critical role in the formation of proper protein folding under both steady-state conditions and inflammatory conditions, but under inflammatory conditions, EBI3 plays an additional role to further augment it.

EBI3 expression is constitutively highest in the placenta but is inducible by infection with EBV in B lymphocytes and activation through Toll-like receptors (TLRs) in dendritic cells (DCs) (32). However, we initially found that EBI3 expression is also induced in naive CD4^+^ T cells by stimulation with plate-bound anti-cluster of differentiation 3 (CD3) and anti-CD28, and we eventually identified a novel role for EBI3 as an intracellular chaperone-like molecule in CD4^+^ T cells (27). By using a T cell-dependent mouse colitis model in which colitis is induced by adoptive transfer of naive CD4^+^ T cells into immunodeficient mice, EBI3-deficient naive CD4^+^ T cells failed to induce colitis with reduced interferon (IFN)-γ production in the intestinal lamina propria lymphocytes. Similarly, IFN-γ production in EBI3-deficient CD4^+^ T cells differentiated under pathogenic T helper 17 (Th17) polarizing conditions with IL-23 *in vitro* was also reduced. However, this effect of EBI3 was not mediated by its culture supernatant, which may contain soluble EBI3 and/or EBI3-related heterodimeric cytokines, suggesting that it was attributed to its intracellular function. During differentiation into pathogenic Th17 cells with IL-23, the expression of one of the IL-23 receptor (IL-23R) subunits, IL-23Rα, but not another IL-23R subunit, IL-12Rβ1, in EBI3-deficient CD4^+^ T cells was selectively decreased at the protein but not mRNA level. Of note, this reduction in IL-23Rα expression was due to synthesis of its misfolded protein and resultant increased proteasomal degradation of IL-23Rα, indicating a chaperone-like role for EBI3 in the ER. Therefore, these results prompted us to consider calnexin, which is a well-known lectin chaperone involved in the proper folding of newly synthesized glycoproteins in the lumen of the ER (38, 39), and whose association with EBI3 in the ER has already been reported (32). As expected, EBI3 augmented IL-23Rα expression *via* binding to calnexin and IL-23Rα in a peptide-dependent but not glycan-dependent manner (27). In the absence of endogenous calnexin, EBI3 failed to augment IL-23Rα expression. Moreover, EBI3 bound to the extracellular region of IL-23Rα, but its binding to the IL-23Rα variant G149R was reduced and therefore EBI3 failed to increase its variant protein expression. Consistent with these results, the IL-23Rα variant G149R, which is associated with protection against the development of inflammatory bowel disease in humans, was recently demonstrated to have its loss of function due to impaired protein stability and resultant decreased signaling by IL-23 (40). Thus, EBI3 plays an important role in further augmenting the expression of IL-23Rα at the protein level in collaboration with calnexin (**Figure 2**).

## EBI3 Facilitates the Proper Protein Folding of p28 to Form IL-27

Given the chaperone-like activity of EBI3, it is highly conceivable and understandable that EBI3 associates with other molecules such as p28, p35, and p19 to form the heterodimers IL-27, IL-35, and IL-39, respectively (**Figure 2**). Indeed, another chaperone-like activity of EBI3 was reported for formation of IL-27 (30, 31). In mice, p28, also designated as IL-30, is secreted and exerts immunoregulatory roles (41), while human p28 is not autonomously secreted due to the difference in one amino acid residue cysteine that is necessary for disulfide bond formation (30). Without EBI3 human p28 is retained and becomes degraded by the ER quality control machinery, whereas the presence of EBI3 facilitates the proper protein folding, leading to efficient secretion of human p28 and formation of IL-27 heterodimer. In mice p28 alone is secreted, and the presence of EBI3 further augments the secretion of mouse p28 and formation of IL-27 heterodimer. Although the formation and secretion of IL-27 heterodimer seem efficient, that does not appear to be the case for the IL-35 heterodimer (42, 43) for unknown reasons that might be related to the chaperone-like activity of EBI3. More detailed analyses on the molecular mechanisms underlying how EBI3 selectively binds to them and forms functional heterodimers are necessary.

## Chaperone-Like Role for EBI3 in Collaboration With Calnexin Under Inflammatory Conditions

As mentioned above, in addition to the role of EBI3 as a subunit of cytokines, EBI3 has an intracellular chaperone-like role to promote the proper folding of target proteins and increase its expression at the protein level by binding to calnexin and its target protein (27). Although EBI3 is predominantly expressed in placenta among normal tissues (32, 44), its expression is induced under pathological conditions including infectious diseases, inflammatory diseases, autoimmune diseases, cancers, and so on as described below. Stimulation of TLRs with lipopolysaccharide (LPS), poly (I:C), and R848 in DCs, monocytes, macrophages, endothelial cells, keratinocytes, and neutrophils induces EBI3 expression through NF-κB activation (8, 45–48). Moreover, EBI3 expression is induced in B cells after infection with EBV, and it is also induced in malaria-specific CD4^+^ T cells by activation through T-cell receptors after infection with malaria, to produce IL-27 (32, 49). It was also recently demonstrated that the infection of macrophages with virulent *Mycobacterium tuberculosis* increases intracellular EBI3 expression *via* its binding to eukaryotic translation elongation factor 1-α1 (eEF1A1), probably to facilitate infection (50). Because it was reported that eEF1A1 exhibits chaperone-like activity (51), the binding of EBI3 to eEF1A1 reduces Lys48-linked ubiquitination of EBI3, leading to accumulation of EBI3, and consequently inhibits caspase 3-mediated apoptosis in macrophages (50). In addition, there are several reports showing that EBI3 seemingly as a self-standing molecule is involved in the pathogenesis of inflammatory diseases including rheumatoid arthritis (52), inflammatory bowel disease (53), systemic sclerosis (54), cardiac inflammation (55), and several cancers (44, 56–58), whereas whether its chaperone-like role is relevant or not remains unknown. Considering that calnexin expression is constitutive but EBI3 expression is thus inducible, calnexin plays a critical role in the formation of proper protein folding under both steady-state and inflammatory conditions, but under inflammatory conditions, EBI3 plays an additional role to augment it. To date, proper protein folding has been mainly discussed independently of steady-state or inflammatory conditions, and there is no special theory to explain it under inflammatory conditions. Therefore, these findings may open an avenue to establish a new concept in which EBI3 plays an important role in promoting the formation of proper protein folding in collaboration with calnexin under inflammatory conditions (**Figure 2**). Further identification of other possible target molecules to which EBI3 binds is necessary for confirmation.

## Cell Surface Expression of ER Chaperones Including EBI3

Many ER chaperones including calreticulin, heat shock proteins, and isomerases translocate to the cell surface *via* binding to other cell surface molecules and are also released into the extracellular space upon ER stress induced by stimuli such as chemicals, ultraviolet irradiation, and microorganisms (59). Some chaperones can then act as danger signals to mount protective immune responses *via* activation of innate and adaptive immune systems. Analogous to these ER chaperones, co-transfection of EBI3 and IL-23Rα expression vectors into a derivative of human embryonic kidney HEK293 cells induces abundant expression of EBI3 on the cell surface, which can be detected by flow cytometry (27). Interestingly, transfection of EBI3 expression vector alone without IL-23Rα significantly induces its cell surface expression, but to a lesser extent, suggesting that EBI3 probably binds to other cell surface molecules that remain to be determined. Further studies are necessary to determine whether the cell surface expression of EBI3 is just a consequence of its chaperone-like activity to increase the expression of target molecules such as IL-23Rα, or whether it has more positive roles such as to function as danger signals, alarmins, find-me signals, eat-me signals, or maybe receptors.

## EBI3 Expression Is Highly Associated With Tumor Growth

Whether the expression of EBI3 predicts a good or poor prognosis for cancer patients remains a subject of debate. This is because, in addition to its intracellular functions, the IL-27 heterodimeric cytokine, which uses EBI3 as one of its subunits, has both antitumor and protumor activities (60) and the IL-35 cytokine mainly has protumor activity by inducing immune suppression, and enhancing tumor angiogenesis and myeloid cell accumulation (61–63). Nevertheless, the upregulation of EBI3 expression is highly associated with tumor progression and metastasis in a variety of cancers including breast (58), gastric (64), lung (44), pancreatic (65), cervical (66), and nasopharyngeal (67) cancers. Nishino et al. (43) previously screened genes encoding transmembrane/secretory molecules that were commonly transactivated in lung cancers by gene expression profile analyses of 120 lung cancers. Interestingly, EBI3 was consequently identified as a molecule whose expression was highly correlated with the poor prognosis of patients with non-small cell lung cancer. Inhibition of endogenous EBI3 expression in lung cancer cells by small interfering RNA markedly reduced their growth, while overexpression of exogenous EBI3 in COS-7 monkey kidney cells greatly enhanced their growth (44). Thus, EBI3 seems to be associated with tumor growth and a highly malignant phenotype of lung cancers. Although the mechanisms underlying the growth-promoting effects of EBI3 remain to be clarified, its chaperone-like activity might be relevant. As one possibility, the EBI3-mediated activation of signaling molecules or receptors may be involved as previously reported for the cell surface Y186-Y190 deletion mutant of gp130, which is retained in the ER due to its association with the chaperone calnexin, and induces autonomous cell growth through STAT3 activation in a ligand-independent manner (68). Although the target molecule of EBI3 remains to be identified, the expression of EBI3 is highly associated with tumor growth.

## p40-Mediated Assembly-Induced Secretion of IL-12 and IL-23

Another β-subunit, p40, which is overexpressed and secreted alone, is necessary for secretion of p35 after forming the IL-12 heterodimer *via* a disulfide bond (69). Newly synthesized p35 in the ribosome fails to form its native structure and is misfolded, forming incorrect disulfide bonds (28). However, the co-expression of p40 inhibits the misfolding and allows secretion of biologically active heterodimer IL-12. Interestingly, misfolding also occurs for p19, and a similar mechanism is applied for the synthesis of IL-23 consisting of p19 and p40 (29). Thus, analogous to EBI3 acting like a chaperone, p40 mediates the assembly-induced folding of p35 and p19 and resultant formation in the ER of their heterodimers IL-12 and IL-23, respectively, which seem to be a common principle for secreting them in this heterodimeric cytokine family.

## Experimental Evidences for Association of EBI3 With p28, p35, and p19 in Primary Cells

Finally, we refer to the issue whether there is any evidence to prove that EBI3 associates with p28, p35, and p19 in primary cells, to form respective natural heterodimers. This is because EBI3 lacks critical cysteine residue necessary for heterodimerization that is present in p40 to form IL-12 and IL-23 (32, 70), and most of studies so far showed the association of each subunit in the cells transfected with each expression vector having different tags. IL-27 is produced in DCs and macrophages after stimulation with stimuli such as LPS and the association between EBI3 and p28 was detected in the culture supernatant by specific sandwich ELISA (6). IL-35 was identified as an inhibitory cytokine that is produced by regulatory T cells and is necessary for maximal suppressive activity (7). In the supernatant of purified mouse regulatory T cells cultured in the absence of stimuli, the association between EBI3 and p35 was clearly detected by immunoprecipitation with anti-p35 antibody followed by western blotting with anti-EBI3 antibody (7). The association between EBI3 and p19 in primary cells was more clearly demonstrated in the following study (10). The presence of natural IL-39 protein in the culture supernatant of purified mouse primary B cells activated with LPS was detected by immunoprecipitation followed by western blotting using respective antibodies. In addition, sandwich ELISA by using anti-19 and anti-EBI3 antibodies as coating and detection antibodies, respectively, clearly detected and determined the concentration of IL-39 in the supernatant. Overall, these evidences could prove but may not completely yet the association of EBI3 with p28, p35, and p19 in primary cells.

## Conclusion and Future Perspectives

Recently, the mutation of another ER chaperone, calreticulin, was demonstrated to be a driver of the development of myeloproliferative neoplasms characterized by the clonal proliferation of hematopoietic stem and progenitor cells (71, 72). This is attributed to constitutive activation of the thrombopoietin receptor, myeloproliferative leukemia protein (MPL), by the mutant calreticulin, resulting in transformation. Surprisingly, the underlying mechanism is so unique that it is currently considered to be due to initial formation of a dimeric or homomultimeric complex of the mutant calreticulin *via* a novel mutant-specific sequence generated by frameshift mutation, and subsequent interaction with immature asparagine-linked glycan for eventual engagement with immature MPL in the ER (73, 74). The complex formed between mutant calreticulin and MPL is then transported to the cell surface, where it induces constitutive activation of the downstream JAK2/STAT5 signaling pathway in an MPL-dependent manner. Whether such a scenario can also be applied to the EBI3-mediated augmentation of tumor growth as mentioned above is an interesting question but remains unknown.

Currently, chaperone-like functions of cytokine subunits and cytokine-like functions of chaperones together with their physiological significance have garnered great interest. A better understanding of the expanding diversity in molecular structures and functions together with their molecular mechanisms and physiological significance would lead to the identification of new heterodimers and associated molecules and the establishment of novel therapeutic strategies by using them as tools and target molecules.

## Author Contributions

All authors contributed to the article and approved the submitted version.

## Funding

This study was supported in part by grants from the Ministry of Education, Culture, Sports, Science, and Technology, Japan.

## Conflict of Interest

The authors declare that the research was conducted in the absence of any commercial or financial relationships that could be construed as a potential conflict of interest.

## Publisher’s Note

All claims expressed in this article are solely those of the authors and do not necessarily represent those of their affiliated organizations, or those of the publisher, the editors and the reviewers. Any product that may be evaluated in this article, or claim that may be made by its manufacturer, is not guaranteed or endorsed by the publisher.
